# Preliminary reference values for electrocardiography, echocardiography and myocardial morphometry in the European brown hare (Lepus europaeus)

**DOI:** 10.1186/1751-0147-51-6

**Published:** 2009-01-30

**Authors:** Agnieszka Noszczyk-Nowak, Józef Nicpoń, Marcin Nowak, Piotr Slawuta

**Affiliations:** 1 Department of Internal and Parasitic Diseases Veterinary Medicine Faculty, Wroclaw University of Environmental and Life Sciences, Wrocław 50-366, Poland; 2 Department of Pathological Anatomy, Veterinary Medicine Faculty, Wroclaw University of Environmental and Life Sciences, Wrocław 50-366, Poland

## Abstract

The study aimed at defining reference values for electrocardiographic (ECG) and echocardiographic parameters as well as macroscopic dimensions of the heart and microscopic dimensions of cardiomyocytes in the European brown hare. The studies were conducted on 30 adult, clinically healthy hares of either sex caught in Poland. ECG and echocardiography were performed supravitally on anaesthetized hares. After euthanasia, gross and microscopic myocardial and cardiomyocyte dimensions were determined. Heart rate amounted to 140 ± 37.5 beats/min, the leading rhythm involved the sinus rhythm. P wave time was 26 ± 5 ms, PQ time was 80 ms, QRS time was 29 ± 3.5 ms, and ST was 97.5 ± 7 ms. Echocardiography determined a left ventricular wall end-diastolic diameter of 8.6 ± 2.0 mm and an intraventricular septum end-diastolic diameter of 5.75 ± 1.0 mm. The thickness of the interventricular septum corresponded to that of the free wall of the left ventricle, a finding consistent with physiological hypertrophy. Preliminary reference values were established for echocardiography. The findings were similar to those obtained at necropsy. The ECG and echocardiographic studies represent the first supravital examination of cardiac function in the hare. The obtained results illustrate adaptation of hare's myocardium to its mode of life. The cardiac findings resemble the athlete's heart syndrome described in humans. The findings may prove useful in further studies on the physiology of the cardio-vascular system in the hare.

## Findings

Studies on the physiology of the European brown hare (*Lepus europaeus*) have focused on organ morphology, blood biochemical parameters, methods of blood sampling, coagulation parameters and cardiovascular disorders [1-6]. Physiological and morphological studies of the heart have not been performed, so the electrocardiographic (ECG) and echocardiographic variables remain unknown. Also, cardiomyocyte morphology remains to be reported.

This study aims at defining reference values related to ECG and echocardiography and to determine gross and microscopic dimensions of the heart in the European brown hare.

The studies were conducted on 30 adult (body weight (BW) 3.2 ± 0.54 kg), clinically healthy hares (10 males and 20 females) out of 96 hares caught in south-eastern Poland. The 30 hares were selected randomly among the 96 hares by selecting every third clinically normal hare. Two hares were omitted from the sampling population due to low age and low BW, respectively. Examination of cardiac morphometry was done in 42 hares, including the 30 hares mentioned above, 8 hares euthanatized due to injuries to extremities, which had developed during transport and 4 hares, which died during the transport. The hares were euthanatized by phenobarbitaladministered intracardially.

The studies obtained consent of the 2^nd ^Local Ethical Commission, No.87/2006 (December 11, 2006). ECG and echocardiography were conducted following anesthesia by a mixture of xylazine (Sedazin, Biowet, Puławy, Poland) 3 mg/kg BW and ketamine (Bioketan, Vetoquinol Biovet, Gorzów Wielkopolski, Poland) 10 mg/kg BW, administered intramuscularly.

**Figure 1:**
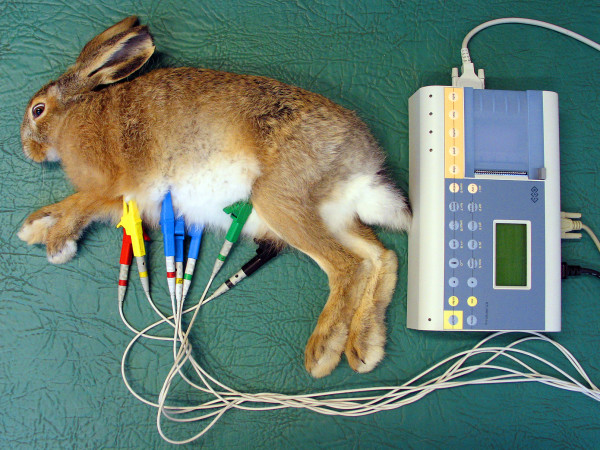
**Sites of electrode placement for electrocardiography in a European brown hare**.

**Figure 2:**
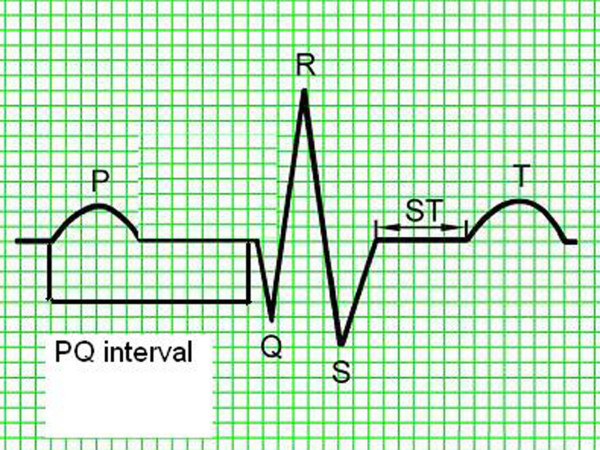
**Schematic presentation of measured electrocardiography parameters**.

**Figure 3:**
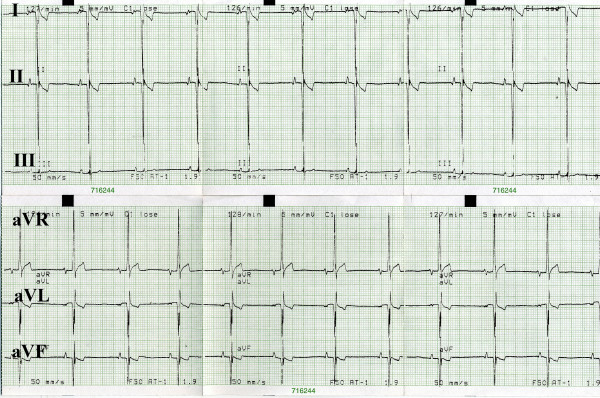
**Electrocardiograph**. Example of an electrocardiography recording in a European brown hare anaesthetized by xylazine and ketamine. Leads I, II, III, aVR, aVL, aVF are shown.

ECG was conducted on animals positioned on their right flank, using a three-channel Sheiler AT-1 apparatus at the pass of 50 mm/s. On extremities the electrodes were placed in line with the generally accepted standards for small animals (Fig. 1) [7]. Amplitudes and duration of P, Q, R, S, T waves, QRS complex, time distances of PQ, QT, ST were measured in the second lead. Duration of P wave was measured from the beginning of the rise to the end of the decrease in the record line. PQ (PR) interval was measured from the beginning of P wave to the beginning of QRS complex. QRS complex was measured from the beginning of Q wave to the end of S wave. QT interval was measured from the beginning of Q wave to the end of T wave. Q wave represents the first negative wave of QRS complex and in several species it is absent from ECG records. R wave represents the first positive wave of QRS complex, the descending arm of which below isoelectric line passes into the negative S wave (Fig. 2). The mean electrical axis (MEA) was calculated on the basis of algebraic sum of QRS complex amplitudes in leads I and III plotted on the coordinate system. The mean electrical axis represents a direction of the resultant electromotive force of the heart and can be applied for diagnosing myocardial hypertrophy or disturbed intraventricular conductance. For every measured ECG parameter its mean value and standard deviation (SD) were calculated and the values allowed for calculation of relevant reference norms (mean ± 2 SD) from 25 cycles. Data from all acral leads (I, II, III, aVR, aVL, aVF) were analyzed to detect disturbances in cardiac rhythm (Fig. 3).

**Figure 4:**
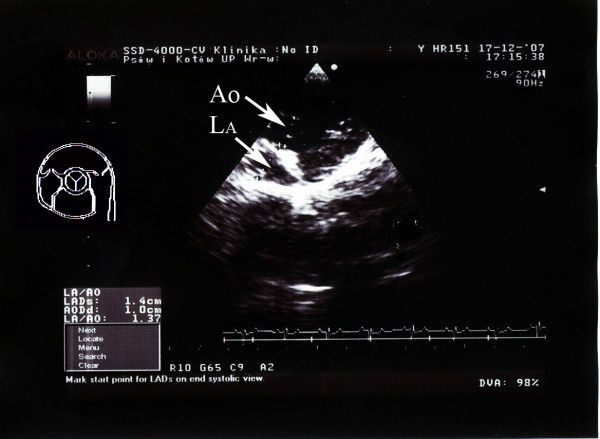
**Echocardiographic image**. Example of an echocardiographic examination (Vascular projection) in a European brown hare anaesthetized by xylazine and ketamine. Aorta: Ao, Left atrium (La).

**Figure 5:**
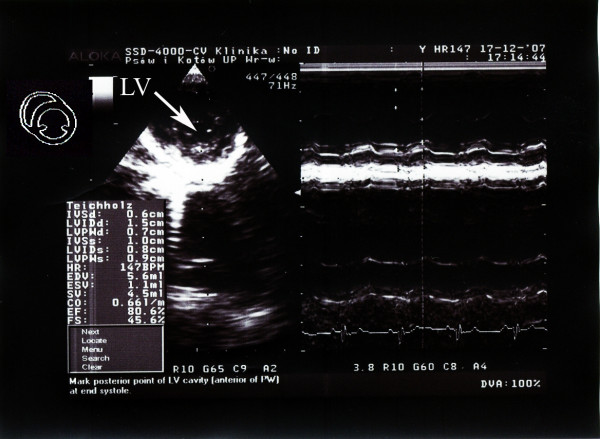
**Echocardiographic image**. Example of an echocardiographic examination in (Parasternal projection in the short axis) a European brown hare anaesthetized by xylazine and ketamine. Left ventricle: LV.

The echocardiographic examination was performed using an Aloka 8000 apparatus equipped with a 7.5–10 Mzh head. Left ventricular end-systolic diameter, left ventricular end-diastolic diameter (LVEDd), left ventricular wall end-diastolic diameter (LWDd) and left ventricular wall end-systolic diameter in diastole as well as intraventricular septum end-diastolic diameter (IVSDd) and intraventricular septum end-systolic diameter were measured. The measurements were taken in parasternal projection in the short axis, from the right hand side, and the probe was placed in the third and fourth intercostal space above the sternum [8]. The measurements allowed for automatic calculation of left ventricle ejection fraction and shortening fraction. Widths of aorta and of left atrium in vascular projection were estimated. For the obtained results, means and standard deviations were calculated and the data provided basis for calculation of reference values (mean ± 2 SD). The relative wall thickness (RWT) was calculated as RWT = IVSDd+LWDd/LVEDd. Examples of echocardiographic images are shown in Figures 4 and 5.

*Post mortem *examination of the cardiac morphometry included measurements of height and width of right and left atrium, right and left ventricle, thickness of the interventricular septum and of the free wall in the left and right ventricle below the atrio-ventricular valves (similar to the site of measurement in echocardiographic examinations), and diameters of atrioventricular and arterial ostia. The locations are illustrated in additional file 1.

Tissue specimens of left ventricular free wall myocardium were fixed for 24 h in buffered 7% formalin, prepared by routine methods for histology, embedded in paraffin and sectioned at 4 μm. Sections were stained by hematoxylin and eosin and subjected to computer-assisted image analysis and morphometric measurements in a setup consisting of a computer connected to an Axiophot optical microscope (Carl Zeiss) equipped with a camera (model CC20P – Videotronic International). The entire set had the potential of recording images and of their digital analysis. The measurements took advantage of MultiScaneBase V 14.02 p software, working in Windows environment. In every of 4 sections 10 optical fields (115 × 150 μm) were examined at 40× magnification. The morphometric analysis included measurements based on transverse as well as cross sections and included number of fibers per optical fields, diameter of cardiomyocytes and length and diameter of cardiomyocyte nuclei (see additional file 2) The obtained mean values with SD and the corresponding calculated reference values of ECG and echocardiographic parameters are presented in Tables 1 and 2, respectively. Gross and microscopic dimensions of the heart are shown in Table 3. Similar gross and microscopic dimensions were obtained independently of the cause of death (spontaneous death *versus *euthanasia).

**Table 1:** Obtained values (mean and standard deviation (SD)) and the corresponding determined reference values (mean ± 2 × SD) for electrocardiographic parameters in European brown hares (N = 30) anesthetized with xylazine and ketamine in the parasternal projection in short axis.

**Parameter**	**Reference value**	**Mean and SD**
Heart rate (beats/min)	100–178	140 ± 37.5

P wave time (ms)	16–36	26 ± 5

P-wave amplitude (mV)	0.14–0.42	0.275 ± 0.07

PQ interval time (ms)	80	80

QRS complex time (ms)	22–36	29 ± 3.5

Q-wave amplitude (mV)	Up to (-)3.2	(-)2.4 ± 0.4

R-wave amplitude (mV)	Up to 5	1.925 ± 1.55

S-wave amplitude (mV)	Up to (-)0.2	0.1 ± 0.05

QT interval time (ms)	100–160	126 ± 10.5

ST interval time (ms)	80–120	97.5 ± 7

T-wave amplitude (mV)	Up to (-) 1.4	(-) 0.6 ± 0.4

Mean electrical axis (^0^)	15–210	97.5 ± 113

**Table 2:** Obtained values (mean and standard deviation (SD)) and the corresponding determined reference values (mean ± 2 × SD) for echocardiographic parameters in European brown hares (N = 30) anesthetized with xylazine and ketamine in the parasternal projection in short axis.

**Parameter (mm)**	**Reference value**	**Mean value and SD**
Left ventricular end-systolic diameter	6–20	13.6 ± 3.7

Left ventricular end-diastolic diameter	3.8 – 13.8	8.8 ± 2.5

Left ventricular wall end-systolic diameter	5.9–13.9	9.9 ± 2.0

Left ventricular wall end-diastolic diameter	6.6–10.6	8.6 ± 2.0

Intraventricular septum end-systolic diameter	2.4–12.4	7.4 ± 2.5

Intraventricular septum end-diastolic diameter	5.55–5.95	5.75 ± 1.0

Left ventricular ejection fraction	46.25–86.25	66.27 ± 9.9

Shortening fraction	18.95–48.75	18.95–48.75

Aorta	4.35 – 11.15	7.75 ± 1.7

Left atrium	11.5 – 18.7	15.12 ± 1.8

**Table 3:** Gross and microscopic dimensions (mean and standard deviation (SD) of the heart of European brown hares (N = 42).

**Parameter**	**Mean value and SD**
Length of the heart (mm)	53.33 ± 9.0

Width of the heart (mm)	39.00 ± 2.82

Height of the right atrium (mm)	15.3 ± 2.73

Width of the right atrium (mm)	14.4 ± 1.37

Ring of tricuspid valve (mm)	16.5 ± 4.7 × 13.16 ± 4.62

Height of the right ventricle (mm)	27.83 ± 3.18

Width of the right ventricle (mm)	27.66 ± 4.84

Myocardial thickness of the right ventricle free wall (mm)	4.66 ± 0.5

Pulmonary artery (mm)	6.16 ± 1.1

Height of the left atrium (mm)	11.16 ± 7.5

Width of the left atrium (mm)	12.3 ± 3.0

Ring of mitral valve (mm)	9.83 ± 3.18 × 11.05 ± 1.55

Height of the left ventricle (mm)	31.83 ± 4.91

Width of the left ventricle (mm)	11.83 ± 1.16

Myocardial thickness of left ventricular free wall (mm)	8.66 ± 1.5

Myocardial thickness of the interventricular septum (mm)	8.16 ± 1.3

Aorta diameter (mm)	6.83 ± 1.47

Number of fibres in the assayed field amounted (mm)	40.52 ± 7.26

Diameter of cardiomyocytes in the ventricle (μm)	20.45 ± 5.06

Length of the cell nucleus (μm)	15.95 ± 2.91

Diameter of the cell nucleus (μm)	4.46 ± 0.63

The performed ECG and echocardiographic studies are the first supravital examinations of cardiac function in the hare. Even when anesthetic drugs were administered no disturbances were observed in cardiac rhythm or cardiac contractility. Anesthesia is needed to perform such studies in wild hares and xylazin-ketamine anesthesia provided a safe anesthesia [2].

The study demonstrated relatively thick ventricular walls and a relatively high ejection fraction thus reflecting the adaptation of hare's myocardium to their mode of life. The findings resemble the athlete's heart syndrome described in humans [9]. The pronounced and frequently repeated exertion leads to concentric hypertrophy of the myocardium without augmentation of cardiac cavities when the main inducing factor involves pressure load in the left ventricle [8]. Such cardiac transformation aims at securing increased stroke volume with preservation of the normal systolic function. The relative wall thickness in humans and in pigs amounts to 0.45 [9,10]. The relative wall thickness of 1.2 ± 0.54 found in this study points to cardiac hypertrophy in hares. No significant differences have been disclosed in thickness of interventricular septum and of free wall in the left ventricle, which indicates physiological hypertrophy [9]. The thickness of the interventricular septum did not differ from that of the free wall in the left ventricle thus indicating physiological hypertrophy [9]. Present studies are, however, of a pioneer character and a larger group of the animals of various ages should be examined. The prominent ventricular myocardium is associated with high values of amplitudes in the QRS ventricular complex and of Q wave in particular. Amplitude of R wave was also substantial, but with high SD. The study showed that hares have cardiomyocytes of a size similar to rabbits [11,12].

*Post mortem *measurements and echocardiographic findings were similar thus demonstrating the usefulness of echocardiography to evaluate the heart of hares. Cardiac measurements were done on left and the right ventricular walls and of interventricular septum just below the atrio-ventricular valves.

The physiological studies based on ECG and echocardiography should be continued in order to verify the preliminarily established reference values.

## Competing interests

The authors declare that they have no competing interests.

## Authors' contributions

ANN carried out of ECG and echocardiographic examinations, calculated the parameters, and drafted the manuscript. JN participated in the drafting and revised the content critically. MN performed the histopathological examinations. PS managed the anesthesia and necropsied the hares. All authors read and approved the final manuscript.

## Supplementary Material

Additional file 1 **Illustrations showing the locations used to measure myocardial dimensions**. a) transverse dimension, b) longitudinal dimension, c) right ventricle diameter, d) intraventricular septum diameter and e) left ventricle diameter. Click here for file

Additional file 2 **Micrographs showing the way cardiomyocytes were measured**. a) cross-section and b) longitudinal section. Hematoxylin and eosin. Obj. ×40 Click here for file

## References

[B1] Marco I, Cuenca R, Pastor J, Velarde R, Lavin S (2003). Hematology and serum chemistry values of the European brown hare. Vet Clin Pathol.

[B2] Nicpon J, Noszczyk-Nowak A, Slawuta P, Kozdrowski (2008). Arterial and venous blood and urine collection techniques in European Brown Hares [in Polish]. Medycyna Wet.

[B3] Nicpon J, Sławuta P, Nicpon J, Noszczyk-Nowak A (2007). Hematological, biochemical and acid-base equilibrium parameters of the European Brown Hare [in Polish]. Medycyna Wet.

[B4] Pikula J, Adam V, Bandouchova H, Beklova M, Horakova J, Horakova H, Kizek R, Krizkova S, Skocovska B, Supalkova V, Svoboda M, Tremi F, Vitula F (2007). Blood coagulation times in the European brown hare (*Lepus europaeus*). Vet Clin Pathol.

[B5] Sargent AP (1974). Spontaneous arteriosclerosis in a brown hare. J Wildl Dis.

[B6] Wright LJ (1975). Spontaneous lesions in the aorta of the common brown hare (*Lepus europaeus*). Vet Pathol.

[B7] Szabuniewicz M, Hightower D, Kyzar JR (1971). The electrocardiogram, vectocardiogram and spatiocardiogram in the rabbit. Can J Comp Med.

[B8] Cheitlin M, Alpert J, Amstrong W (1997). ACC/AHA guidelines for the clinical application of echocardiography. Circ.

[B9] Rich BS, Havens SA (2004). The athletic heart syndrome. Curr Sports Med Rep.

[B10] Noszczyk-Nowak A, Pasławska U, Zyśko D, Gajek J, Nicpoń J, Rabczyński J, Skrzypczak P (2007). Cardiac hypertrophy induced by administration oral of L-thyroxine in growing pigs [in Polish]. Medycyna Wet.

[B11] Loughrey CM, Smith GL, MacEachern KE (2004). Comparison of Ca^2+ ^release and uptake characteristics of the sarcoplasmic reticulum in isolated horse and rabbit cardiomyocytes. Am J Physiol Heart Circ Physiol.

[B12] Zak R (1974). Development and proliferative capacity of cardiac muscle cells. Circ Res.

