# Supplementary figures and images for: Preliminary reference values for electrocardiography, echocardiography and myocardial morphometry in the European brown hare (Lepus europaeus)
Source: Acta Vet Scand. 2009 Jan 30;51(1):6. doi: 10.1186/1751-0147-51-6 (PMC2646734; doi:10.1186/1751-0147-51-6)

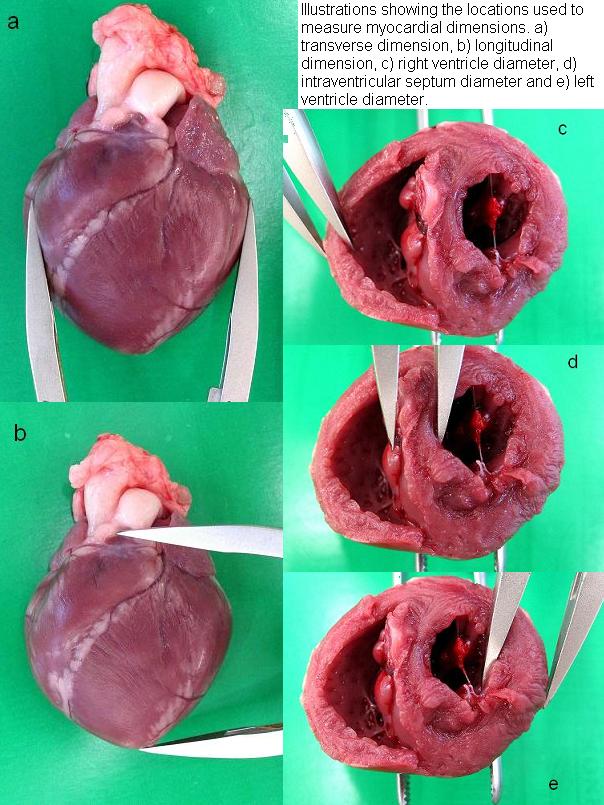

Supplement: Additional file 1 — Illustrations showing the locations used to measure myocardial dimensions. a) transverse dimension, b) longitudinal dimension, c) right ventricle diameter, d) intraventricular septum diameter and e) left ventricle diameter. [file 1751-0147-51-6-S1.jpeg]

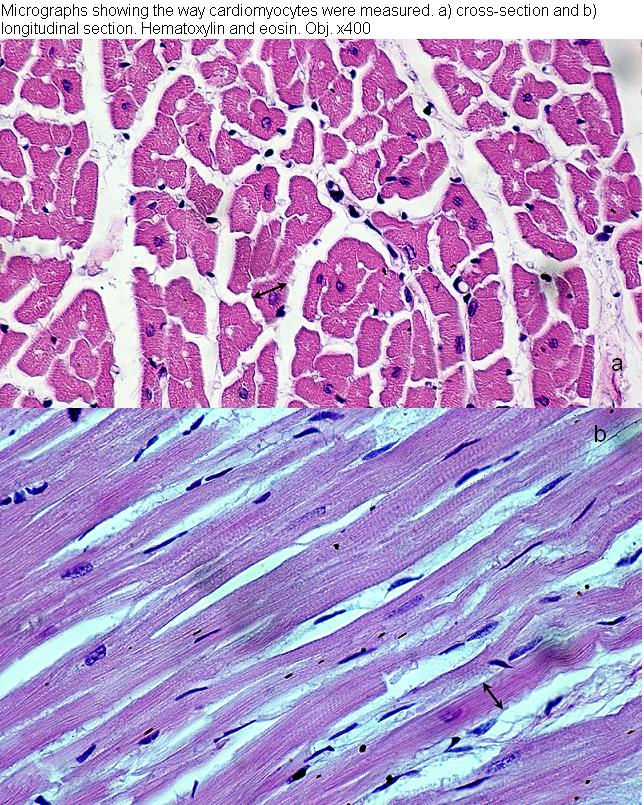

Supplement: Additional file 2 — Micrographs showing the way cardiomyocytes were measured. a) cross-section and b) longitudinal section. Hematoxylin and eosin. Obj. ×40 [file 1751-0147-51-6-S2.jpeg]
